# Identifying gaps in the HIV treatment cascade in Africa: a model comparison study

**DOI:** 10.1016/S2214-109X(25)00121-4

**Published:** 2025-06

**Authors:** Loveleen Bansi-Matharu, Haroon Moolla, Daniel T Citron, John Stover, Michael Pickles, Rowan Martin-Hughes, Marie-Claude Boily, Rose Nyirenda, Edinah Mudimu, Debra ten Brink, Leigh F Johnson, Owen Mugurungi, Valentina Cambiano, Dobromir Dimitrov, Jenny Smith, Robert Glaubius, Issac Taramusi, Amon Mpofu, Andrew Phillips, Anna Bershteyn

**Affiliations:** UCL Centre for Clinical Research, Epidemiology, Modelling and Evaluation, Institute for Global Health, University College London, London, UK; Centre for Infectious Disease Epidemiology and Research, University of Cape Town, Cape Town, South Africa; Department of Population Health, New York University Grossman School of Medicine, New York, NY, USA; Avenir Health, Takoma Park, MD, USA; Department of Infectious Disease Epidemiology, Imperial College London, London, UK; Burnet Institute, Melbourne, VIC, Australia; MRC Centre for Global Infectious Disease Analysis, School of Public Health, Imperial College London, London, UK; Department of HIV and AIDS, Ministry of Health Malawi, Lilongwe, Malawi; Department of Decision Sciences, University of South Africa, Pretoria, South Africa; Burnet Institute, Melbourne, VIC, Australia; Centre for Infectious Disease Epidemiology and Research, University of Cape Town, Cape Town, South Africa; Ministry of Health & Child Welfare, Harare, Zimbabwe; UCL Centre for Clinical Research, Epidemiology, Modelling and Evaluation, Institute for Global Health, University College London, London, UK; Public Health Sciences Division, Fred Hutchinson Cancer Center, Seattle, WA, USA; UCL Centre for Clinical Research, Epidemiology, Modelling and Evaluation, Institute for Global Health, University College London, London, UK; Center for Modeling, Planning and Policy Analysis, Avenir Health, Glastonbury, CT, USA; UNAIDS Country Office Zimbabwe, Harare, Zimbabwe; National AIDS Council, Harare, Zimbabwe; UCL Centre for Clinical Research, Epidemiology, Modelling and Evaluation, Institute for Global Health, University College London, London, UK; Department of Population Health, New York University Grossman School of Medicine, New York, NY, USA

## Abstract

**Background:**

Although HIV incidence has considerably decreased in eastern, central, and southern Africa, new HIV infections continue to be a major public health challenge in the region. We aimed to investigate where in the HIV treatment cascade new transmissions are occurring in Malawi, Zimbabwe, and South Africa (the three countries involved in the Modelling to Inform HIV Programmes in Sub-Saharan Africa project).

**Methods:**

In this model comparison study, we used six well described and independently calibrated HIV transmission dynamics models that have been used to inform HIV policy in Africa (Optima HIV, EMOD, Goals, Thembisa, PopART-IBM, and HIV Synthesis) to estimate and predict the proportion of annual new HIV transmissions attributable to people living with HIV who are undiagnosed, have been diagnosed but have not yet started antiretroviral therapy (ART), are receiving ART, and have interrupted ART in Malawi, Zimbabwe, and South Africa from 2010 to 2040 stratified by the age and sex of the individual acquiring HIV.

**Findings:**

Despite the different model structures and underlying assumptions, the six models were well aligned in relation to key HIV epidemic characteristics (including population estimates and HIV prevalence) in each of the three settings. There was, however, considerable variation in the predicted number of new infections, particularly in Malawi and Zimbabwe where this number ranged from fewer than 10 000 new infections to over 30 000 new infections in 2024. Most model results suggested that the mean age of HIV acquisition has been increasing since 2000, with men acquiring HIV at an older age than women in all three settings. All models attributed fewer than 5% of transmissions to individuals who had been diagnosed but had not yet started ART. In Malawi, the proportion of transmissions attributable to undiagnosed people with HIV in 2024 ranged from 33·3% to 75·3% across the models, and transmissions attributable to individuals who had experienced interrupted treatment ranged from 8·4% to 20·1%. In Zimbabwe, the proportion of transmissions attributable to undiagnosed individuals in 2024 ranged from 29·8% to 64·6% across the models and the proportion of transmissions attributable to individuals who had interrupted treatment ranged from 4·7% to 21·5%. In South Africa, 21·8–46·4% of transmissions in 2024 were attributable to undiagnosed individuals and 27·6–58·9% of transmissions were attributable to individuals who had interrupted treatment.

**Interpretation:**

Across the three study settings, a substantial proportion of new HIV transmissions were attributable to undiagnosed individuals and people who have received interrupted ART, reinforcing the importance of continuing HIV testing and ART re-engagement and retention interventions.

**Funding:**

The Bill & Melinda Gates Foundation.

## Introduction

HIV incidence in eastern, central, and southern Africa (ECSA) has declined considerably over the past 20 years, with new infections falling by 49–57% between 2010 and 2022.^1^ This trend is due to several factors: earlier declines in incidence were probably due to changes in sexual behaviour and condom use,^2^ whereas more recent declines are the result of large-scale HIV programmes with proactive HIV testing, easier access to antiretroviral therapy (ART), and the promotion of HIV prevention (including pre-exposure prophylaxis and voluntary medical male circumcision).^3^

Despite these gains, new HIV infections continue to be a major public health challenge in the ECSA region. In 2022, UNAIDS estimated 450 000 new HIV infections in eastern and southern Africa and 190 000 new infections in western and central Africa.^4^ An important parallel effort to the scale-up of HIV prevention therefore includes the identification of sources of new HIV acquisitions and redoubled efforts to reach these individuals with transmission-reducing interventions. Transmission potential arises from a combination of biological factors (particularly high HIV viral loads due to inconsistent adherence to antiretroviral therapy [ART] or, in some cases, drug resistance—although resistance has largely diminished as an issue with greater use of dolutegravir-based treatment regimens)^5^ and behavioural factors, such as sexual activity and incorrect or inconsistent condom use.

Understanding sources of transmission along the so-called HIV treatment cascade (ie, the proportion of infections that are directly transmitted by people living with HIV who are undiagnosed, diagnosed but not receiving treatment, receiving treatment that is suboptimally effective, or have interrupted treatment) is of particular policy interest. Initial population-based HIV impact assessment (PHIA) surveys in Malawi, Zambia, and Zimbabwe suggest that half of people newly diagnosed with HIV are diagnosed late,^6^ increasing the likelihood of further transmission when undiagnosed. PHIA data have also been used to directly understand potential transmission risk by calculating a range of measures, including adjusted prevalence ratios of self-reported high-risk behaviours, across treatment cascade stages.^7^ Edun and colleagues^7^ found that in 14 surveys across the ECSA region, people who were undiagnosed and (to a lesser but still considerable extent) people who had been diagnosed but had not yet received treatment contributed most to HIV transmission. On the other hand, some evidence suggests that ART-experienced individuals who receive interrupted treatment contribute a growing proportion of HIV transmissions and HIV-related illnesses and deaths.^8–10^

By better understanding which stages of the treatment cascade have contributed most to transmission at different times throughout the HIV pandemic, policy makers can optimise efforts to strengthen HIV treatment as prevention. Modelling can add further insights to empirical data and can be used to predict where in the HIV treatment cascade most transmissions are likely to come from in the future.

This model comparison study aimed to investigate where in the HIV treatment cascade transmissions occur in Malawi, Zimbabwe, and South Africa (the three countries involved in the Modelling to Inform HIV Programmes in Sub-Saharan Africa project) with six well established, independent, calibrated HIV transmission dynamics models.

## Methods

### Contributing models

This model comparison study compared results from six well described HIV transmission models that have been used to inform HIV policy in Africa: Optima HIV, EMOD, Goals, Thembisa, PopART-IBM, and HIV Synthesis. The key features of the six models are summarised in the table.

The six models have different structures and underlying assumptions and each model was independently parameterised and calibrated to specific settings.^11–16^ We did not attempt to align these assumptions and parameters for this study as we wanted to ensure that the outputs reflected a wide range of modelling approaches and assumptions. However, several iterations of outputs generated before the ones presented in this Article were discussed by the modelling teams, and the model assumptions were reflected on and modified where possible to explain differences in results.

The models assumed different cutoffs for the undetectable viral load below which HIV was assumed to be untransmissible. For example, Optima HIV assumed that undetectable equalled untransmissible (U=U) hence there were no transmissions if the viral load of the source partner was <400 copies per mL, whereas in Goals, U=U was assumed for individuals with viral loads <1000 copies per mL. In HIV Synthesis, U=U was assumed for viral loads <200 copies per mL. EMOD and PopART-IBM did not assume U=U among people with undetectable viral loads, but more than 90% of this population was assumed to be receiving effective ART and were assumed to be unable to transmit HIV.

The models also varied in relation to how they established the proportion of people with HIV receiving ART with an undetectable viral load. In Optima HIV, HIV Synthesis, and PopART-IBM, viral suppression was based on the duration of and adherence to treatment (with the addition of drug resistance in HIV Synthesis). In EMOD, 8% of transmissions from individuals currently receiving ART were attributable to individuals with viral loads <1000 copies per mL. In Thembisa, the distribution of viral loads in individuals receiving ART was represented with a reverse Weibull distribution, with the shape parameter established from International Epidemiology Databases to Evaluate AIDS data^17^ and the mean calculated from South African HIV programme data.^12^ In Goals, PHIA and programme viral suppression data were used to set viral suppression levels over time in each country (appendix p 159).

### Model outputs

Each model provided outputs on one or more of the three settings considered. Goals, Optima HIV, and HIV Synthesis provided outputs for Malawi, Zimbabwe, and South Africa; EMOD provided outputs for Malawi; PopART-IBM provided outputs for Zimbabwe; and Thembisa provided outputs for South Africa.

For each setting, modelling groups output the annual number of new HIV transmissions for each year from 2010 to 2040 stratified by the treatment cascade state of the source partner from whom HIV was acquired and the age and sex of the individual acquiring HIV. The inferred treatment cascade state of the source partner was categorised as: undiagnosed; diagnosed and ART-naive (ie, never started treatment); receiving ART; or having ART interrupted at the time of transmission. These categories were further broken down according to the time that had passed since the source partner first started ART or reinitiated ART after interruption (<6 months or >6 months), the source partner’s viral load at transmission, and the source partner’s CD4 cell count (at the start of ART, at reinitiation of ART after interruption, and at time of transmission), depending on whether the models were able to record this information (appendix p 17). Of the six models contributing to this analysis, EMOD and PopART-IBM were the only network-based models that could explicitly model HIV acquisition from a specific individual. All other models inferred the treatment status of the person transmitting HIV on the basis of the cascade stage of the simulated HIV-positive population or by running counterfactual scenarios in which transmission rates were reduced to zero at different disease stages and calculating the difference in new overall annual HIV transmission.

The primary output of the proportion of infections attributable to specific treatment cascade states of source partners between 2000 and 2040 was calculated. Further outputs categorised by viral load and CD4 cell count can be found in the appendix (p 10).

### Role of the funding source

The funder of the study had no role in study design, data analysis, data interpretation, or writing of the report.

## Results

**Figure 1:**
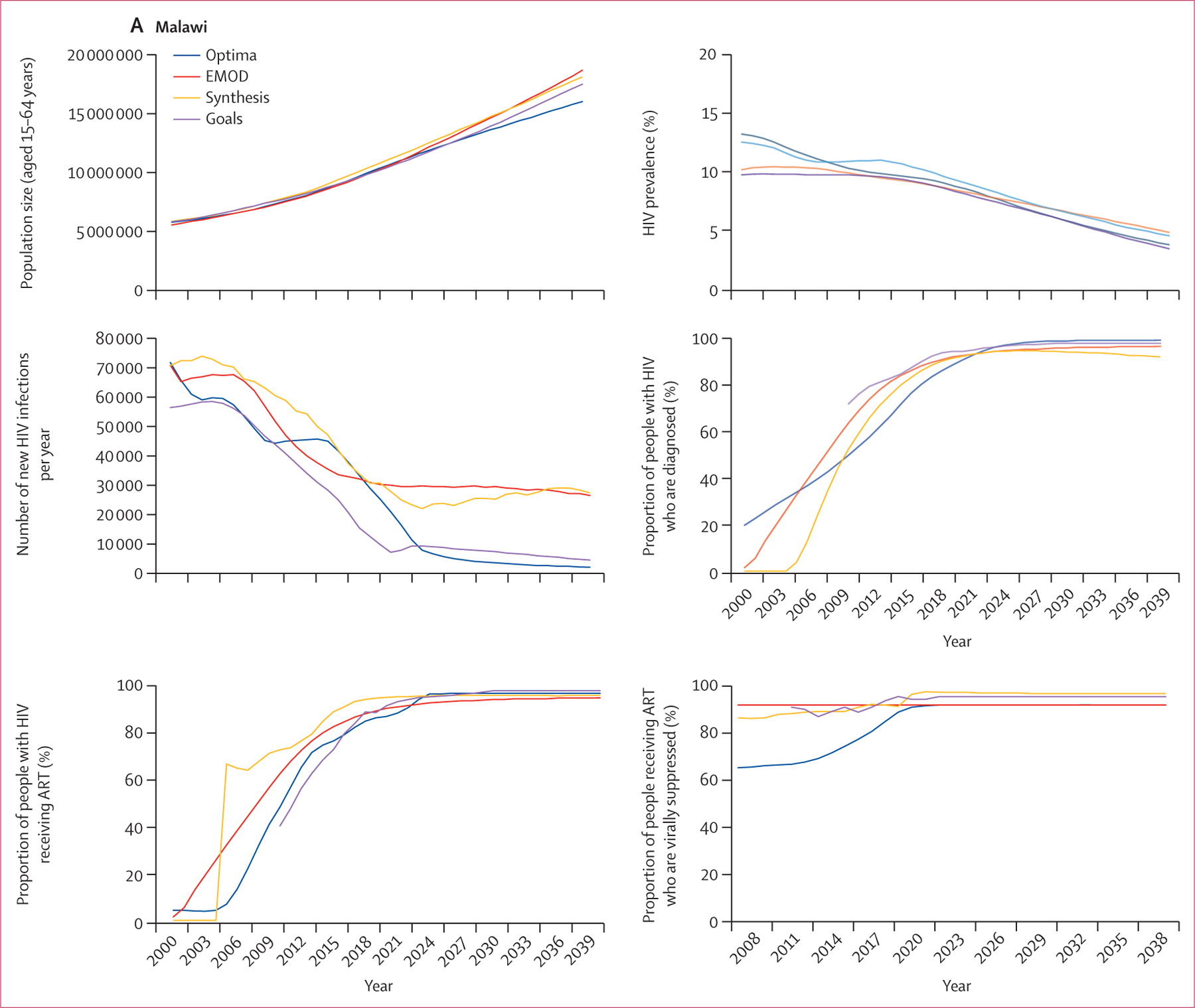

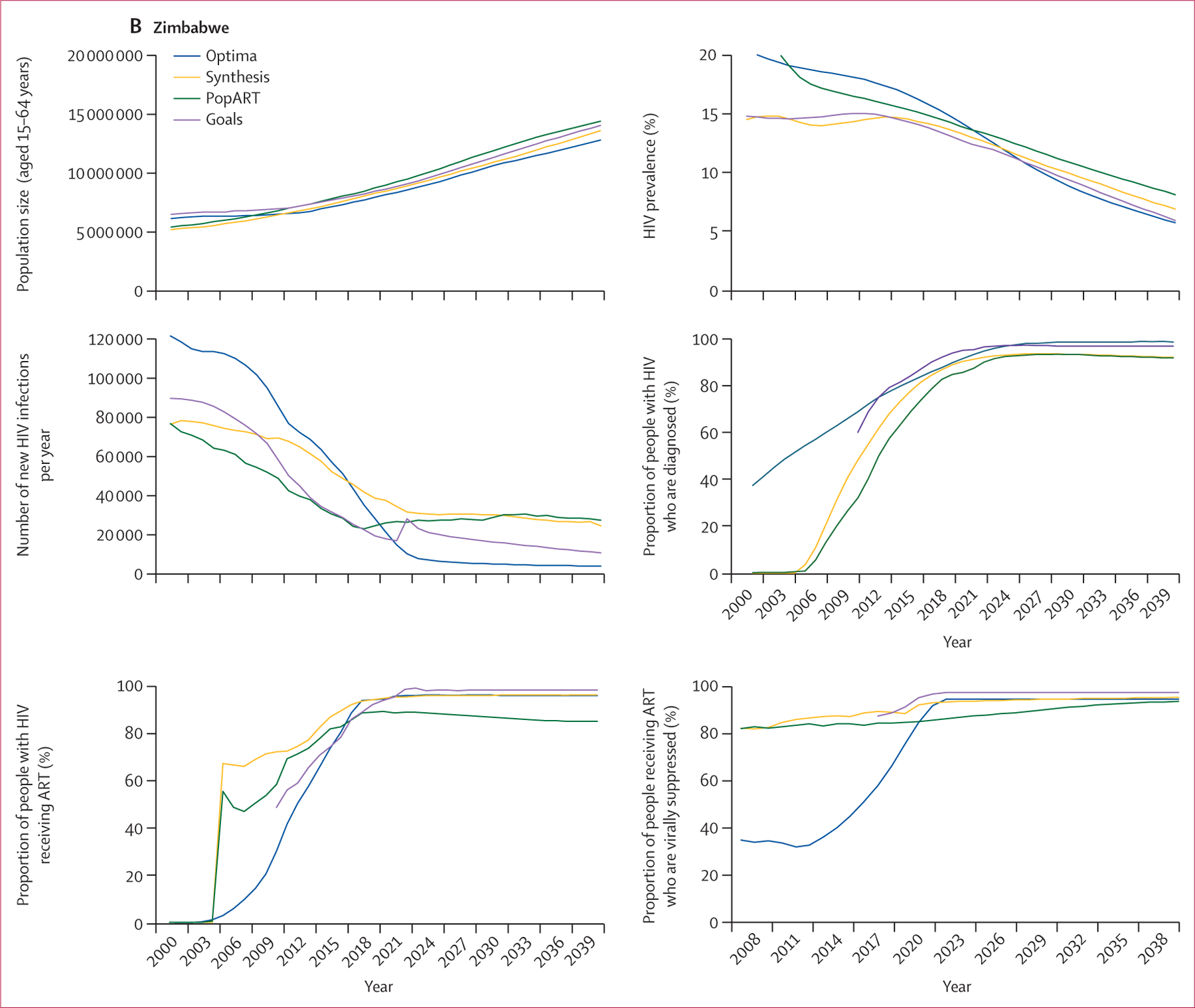

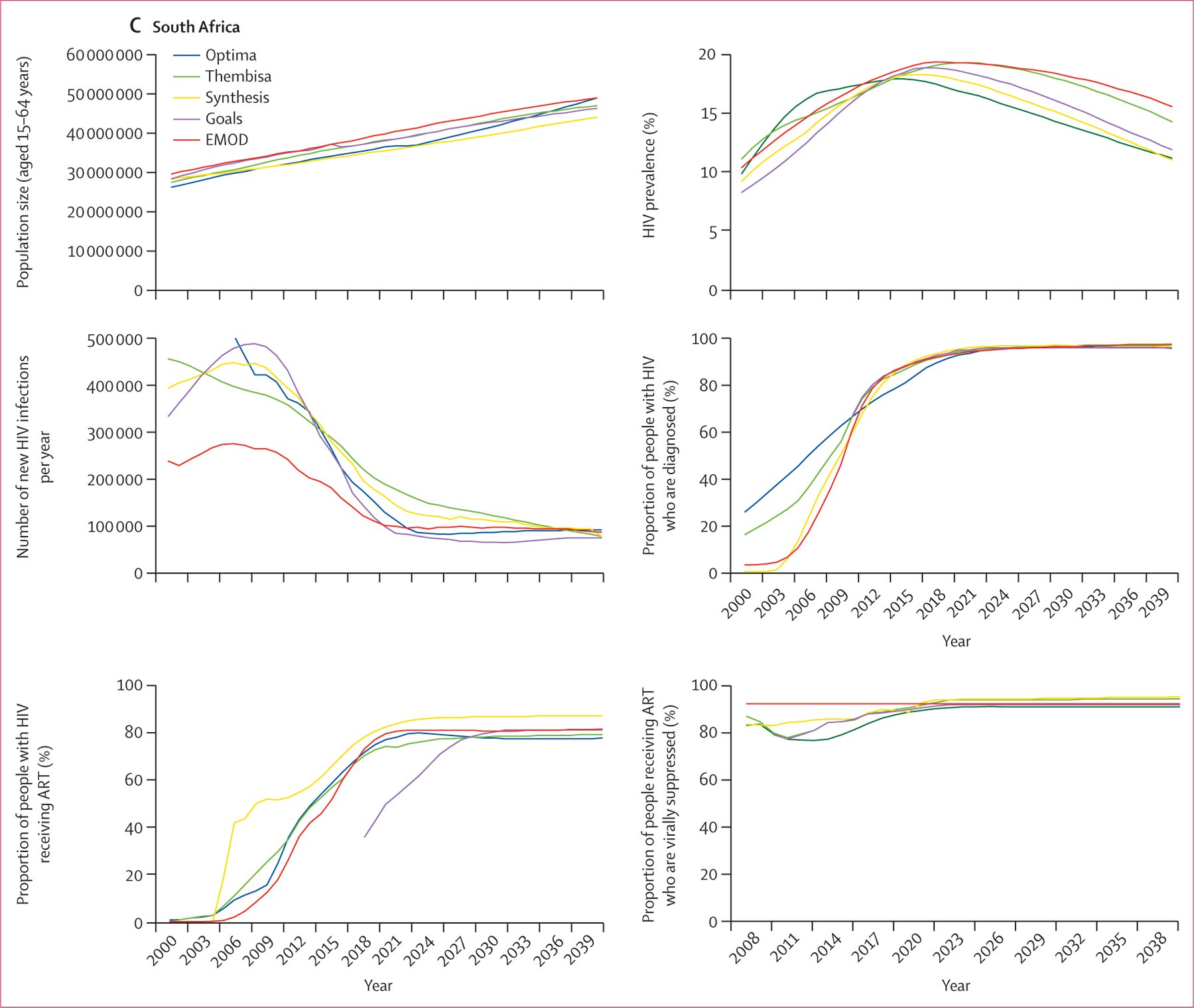
HIV epidemic characteristics for adults aged 15–64 years in Malawi (A), Zimbabwe (B), and South Africa (C) ART=antiretroviral therapy.

The key HIV epidemic trajectories for each of the three countries are shown in figure 1. Overall, despite the different model structures and underlying assumptions, the models were well aligned in relation to general population estimates and HIV prevalence within the different settings, especially from 2020 onwards. There was, however, considerable variation in the predicted number of new infections, particularly in Malawi and Zimbabwe where this number ranged from fewer than 10 000 new infections to over 30 000 new infections in 2024. The modelled proportions of people with HIV who are diagnosed, receiving ART, and virally suppressed were generally similar for each country, although these proportions reached close to 100% according to some models but not in others. In Zimbabwe, the proportion of people living with HIV who were diagnosed was projected to remain constant after 2024 according to Optima HIV and Goals but was projected to decline according to HIV Synthesis and PopART-IBM, partly because both HIV Synthesis and PopART-IBM assumed that the total population would increase over time whereas the number of HIV tests would remain constant (figure 1B). In Zimbabwe, the proportion of people receiving ART among those diagnosed was lower in the PopART-IBM outputs than in those from Optima HIV or HIV Synthesis, probably due to the higher treatment dropout rates assumed in PopART-IBM.

**Figure 2:**
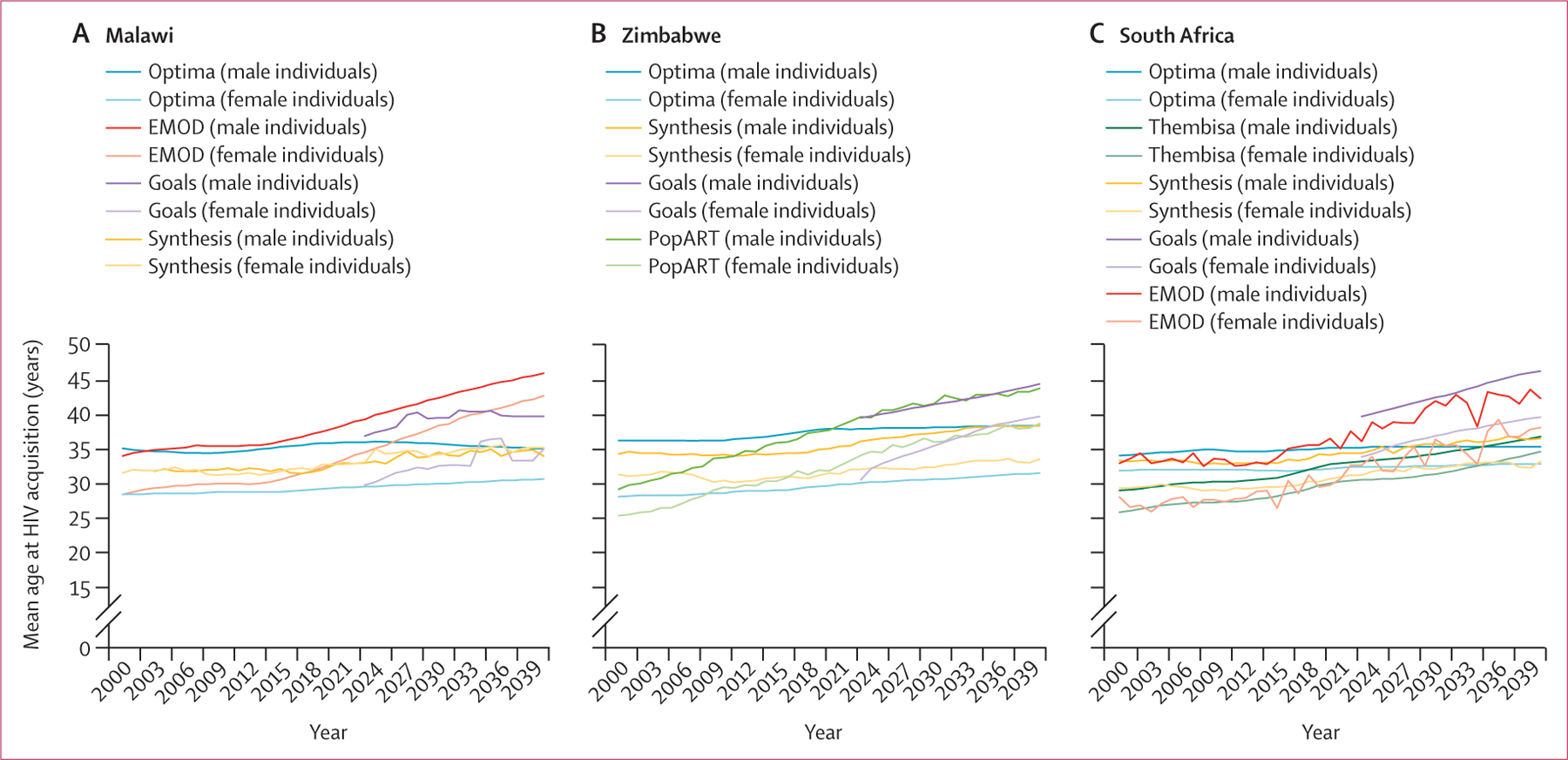
Mean age at HIV acquisition in Malawi, South Africa, and Zimbabwe

Most model results suggested that the mean age of HIV acquisition has been increasing since 2000, with men acquiring HIV at an older age than women in all three settings (figure 2). In Malawi, the Optima HIV model projected that age at acquisition had remained stable at approximately 35 years for men and 30 years for women, whereas EMOD projected an increase in age at acquisition from 40 years in 2024 to 46 years in 2040 for men and from 35 years in 2024 to 43 years in 2040 for women (figure 2A). In Zimbabwe, all models projected an increase over time, although this increase was less pronounced according to Optima HIV (figure 2B). Age at acquisition in 2024 ranged from 36 years (HIV Synthesis) to 40 years (Goals and PopART-IBM) for men and from 30 years (Optima HIV) to 35 years for women (PopART-IBM). In South Africa, an increase in age at acquisition was projected by most models between 2024 and 2040 among both men and women, although Optima HIV projected a stable trend from 2024 onwards at around age 35 years in men and age 33 years in women (figure 2C).

**Figure 3:**
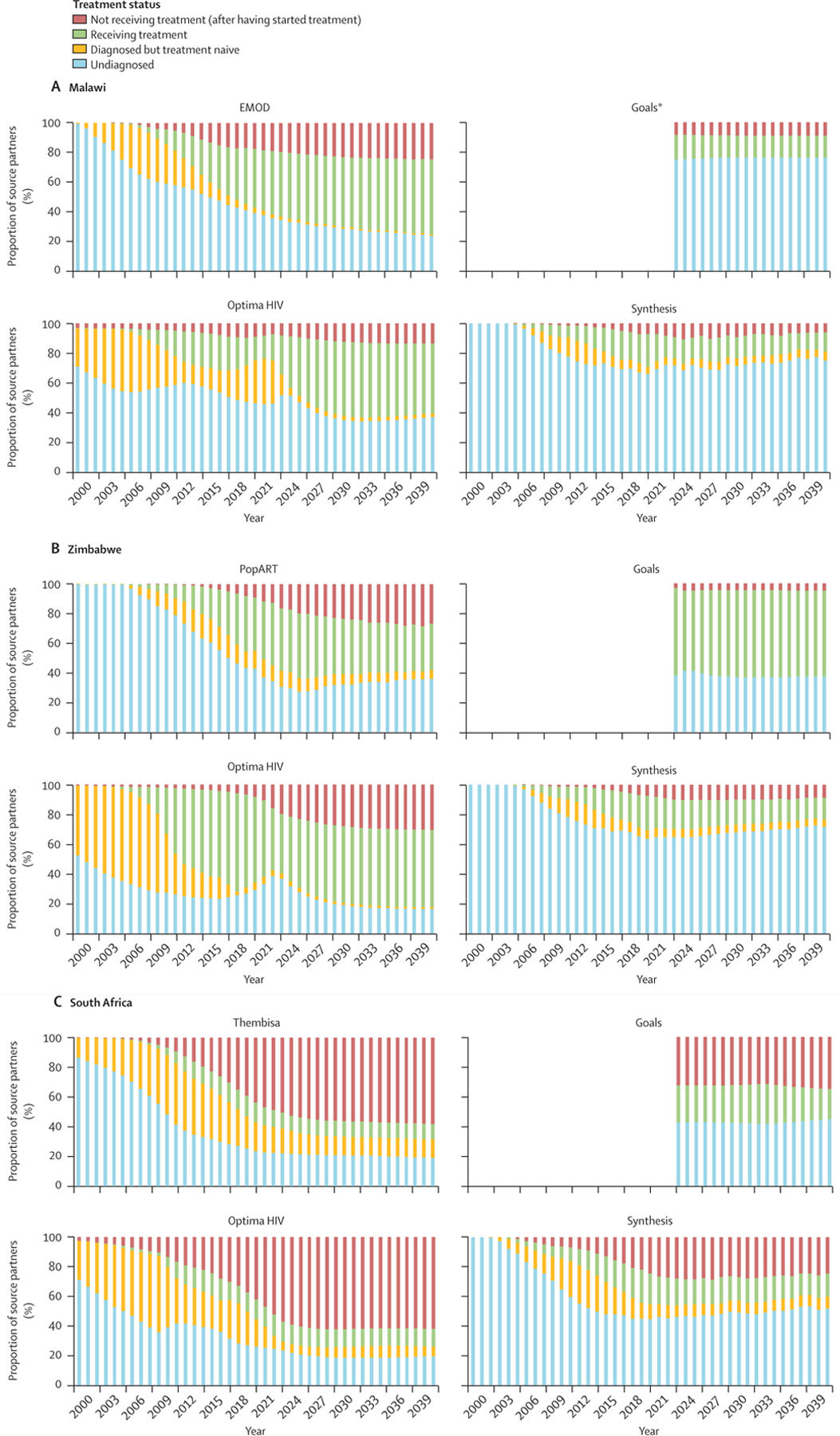
Treatment status of source partners in Malawi (A), Zimbabwe (B), and South Africa (C) by model (A) Number of new infections in 2024: 30 582 infections in EMOD; 10 120 infections in Goals; 8459 infections in Optima HIV; and 22 689 infections in HIV Synthesis. *Goals did not differentiate between people who had been diagnosed and were treatment naive and those who had started treatment but had had it interrupted; these individuals were all classified as people not receiving treatment. (B) Number of new infections in 2024: 27 581 infections in PopART-IBM; 21 384 infections in Goals; 6772 infections in Optima HIV; and 30 536 infections in HIV Synthesis. (C) Number of new infections in 2024: 164 252 infections in Thembisa; 95 513 infections in Goals; 101 852 infections in Optima HIV; and 138 872 infections in HIV Synthesis.

Patterns in the treatment status of source partners are shown in figure 3. The proportions were calculated with the number of new HIV infections as the denominator, which was variable (as shown above). These results should therefore be interpreted in line with the HIV epidemic characteristics described earlier (figure 1).

In Malawi (modelled by four models: EMOD, Optima HIV, Goals, and HIV Synthesis) in 2024, the proportion of HIV transmissions attributable to undiagnosed people with HIV ranged from 33·3% to 75·3% across the models (figure 3A). The proportion of transmissions attributable to people with HIV receiving ART ranged from 16·3% to 44·3%, and the proportions attributable to people living with HIV who had interrupted treatment ranged from 8·4% to 20·1%. All models projected that less than 5% of transmissions were attributed to people with HIV who had been diagnosed but had not yet started ART. EMOD projected a decreasing proportion of transmissions attributable to undiagnosed individuals with HIV over time (from 33·3% in 2024 to 23·7% in 2040; figure 3A). Although Optima HIV also projected a small decrease in the proportion of transmissions attributable to undiagnosed individuals with HIV between 2000 and 2008, this proportion remained stable afterwards (at approximately 37·8%). According to Goals and HIV Synthesis, the proportions of transmissions attributable to undiagnosed individuals remained stable over time but were much higher than in the Optima HIV and EMOD outputs (75·3% of transmissions in Goals and 68·5% of transmissions in HIV Synthesis in 2024). The proportion of transmissions attributable to individuals receiving ART was projected to increase over time by EMOD and Optima HIV, albeit by different magnitudes: in 2024, 46·1% of transmissions were attributable to individuals receiving ART in the EMOD outputs versus 34·8% in the Optima HIV outputs (figure 3A), with both proportions expected to continue to increase up to 2040 when they reach similar levels according to both models. Goals and HIV Synthesis projected stable proportions of transmissions attributable to individuals receiving ART over time, and considerably lower proportions (16·3% of transmissions in 2024) than those projected by EMOD and Optima HIV (figure 3A).

In Zimbabwe (modelled by four models: PopART-IBM, Optima HIV, HIV Synthesis, and Goals) in 2024, the proportion of transmissions attributable to undiagnosed individuals ranged from 29·8% to 64·6%, the proportion attributable to individuals receiving ART ranged from 19·5% to 54·2%, and the proportion attributable to individuals who had interrupted treatment ranged from 4·7% to 21·5% (figure 3B). As in Malawi, the proportion of transmissions attributed to individuals who were diagnosed but had not started ART decreased over time and remained low across the models. From 2024 onwards, transmissions attributable to undiagnosed individuals were stable according to Optima HIV and Goals and slightly increased according to PopART-IBM and HIV Synthesis, albeit to different magnitudes. The proportion of transmissions attributable to individuals receiving ART was projected to increase modestly by Goals and Optima HIV but projected to decrease by PopART-IBM and Synthesis (figure 3B).

In South Africa (modelled by four models: Thembisa, Optima HIV, HIV Synthesis, and Goals), 21·8% to 46·4% of transmissions were attributable to undiagnosed individuals and appeared to remain stable from 2024 onwards (figure 3C). As seen in Zimbabwe and Malawi, the proportion of transmissions attributed to diagnosed individuals who had not yet started ART declined over time, although the magnitude of this decline varied between models, and in 2024 ranged from 5·6% to 15·5%. In 2024, transmissions attributable to individuals receiving ART remained stable over time (range 10·2–24·6%) and transmissions attributable to individuals who had interrupted treatment ranged from 27·6% to 58·9% (figure 3C)—higher than in Malawi and Zimbabwe.

The proportion of transmissions attributable to people living with HIV in each of the status categories (eg, of individuals who were undiagnosed, the proportion who transmitted HIV) is shown in the appendix (p 16).

The distribution by treatment status of source partners transmitting HIV stratified by the age of the individual acquiring HIV is shown in the appendix (p 3). Within each setting, differences in the source of transmission between age groups were minor according to Optima HIV, PopART-IBM, and HIV Synthesis but were more substantial according to EMOD, Goals, and (to a lesser extent) Thembisa. EMOD projected that the proportion of transmissions attributable to undiagnosed individuals in Malawi was highest among individuals acquiring HIV aged 15–24 years and lowest among people acquiring HIV older than 35 years. Similarly, transmissions attributable to source partners receiving ART or those who had interrupted treatment was lowest among people acquiring HIV aged 15–24 years and highest among people acquiring HIV older than 35 years. Although Goals did not project differences in the treatment status of source partners with HIV in different age groups in Malawi, differences were projected in Zimbabwe and South Africa (similar to those projected by EMOD); the proportion of infections attributable to undiagnosed individuals was higher in younger age groups (ie, aged 15–24 years) and the proportion of infections attributable to source partners receiving ART was higher in older age groups (ie, older than 35 years). Similar patterns were seen in South Africa with Thembisa, albeit to a lesser degree.

The treatment status of source partners transmitting HIV stratified by the sex of the person acquiring HIV is shown in the appendix (p 5). In Malawi, although most models found historical differences between the sexes with regards to the treatment status of source partners (eg, EMOD projected that female HIV acquisitions before 2020 were more likely to be attributable to individuals who were diagnosed but had not yet started ART than male acquisitions; in 2010, 38·7% of female acquisitions were attributable to individuals who had been diagnosed but who had not started treatment *vs* 17·9% of male acquisitions), these differences generally subsided after 2020. In Zimbabwe, projections by PopART-IBM and Optima HIV were similar for men and women, but both Goals and HIV Synthesis projected that a higher proportion of acquisitions in women were attributable to an undiagnosed source partner than in men. Goals also found that acquisitions in women were less likely to be attributable to a source partner receiving ART than acquisitions in men. In South Africa, projections were similar between the sexes, although some differences were seen in relation to the proportion of partners who had interrupted treatment (eg, Thembisa projected a higher proportion of acquisitions in women attributable to a source partner who had interrupted treatment than in men [54·6% of acquisitions in women *vs* 48·1% of acquisitions in men in 2024]).

Transmissions stratified by the viral load of the source partner are shown in the appendix (p 10). In Optima HIV, zero transmissions attributable to individuals receiving ART with an undetectable viral load were assumed. In EMOD, 8% of transmissions were attributable to those with an undetectable viral load. In HIV Synthesis, <5% of transmissions were attributable to source partners with a viral load below 1000 copies per mL (ie, these transmissions were from people with viral loads of 200–1000 copies per mL) while receiving ART. 40% of new transmissions in PopART-IBM were attributable to individuals with an undetectable viral load in 2023; this was projected to increase to up to 58% by 2040. These projections were higher than those seen in other models since PoPART-IBM did not assume U=U, and up to 10% of people living with HIV with viral loads <1000 copies per mL could potentially transmit HIV.

Optima HIV was able to further stratify the treatment status of source partners according to CD4 cell counts at the time of transmission. Among source partners receiving ART in Malawi, around 50% had CD4 counts <200 cells per μL at the time of transmission in 2023 (appendix p 11). This proportion was lower in Zimbabwe, where around a third of people transmitting HIV had CD4 counts <200 cells per μL at the time of transmission in 2023 (and this proportion declined further to around a fifth in 2040). In South Africa, approximately 40% of individuals receiving ART and transmitting HIV were predicted to have CD4 counts <200 cells per μL at the time of transmission in 2023, declining to around 30% in 2040. There was also a considerable difference according to sex in Malawi (although this was not seen in Zimbabwe or South Africa; appendix p 12); the source partners of men acquiring HIV were less likely to have a CD4 count <200 cells per μL at the time of transmission than the source partners of women acquiring HIV. EMOD (with outputs for Malawi) and PopART-IBM (with outputs for Zimbabwe) were able to stratify the treatment status of source partners according to CD4 cell counts at initiation and re-initiation of ART after interruption (appendix p 12). Among transmissions attributable to individuals who had received ART for fewer than 6 months since first initiation of treatment, the majority of source partners had CD4 counts <200 cells per μL at ART initiation in Malawi from 2023 to 2040 (range 70·0–89·8% depending on age). Acquisitions in men were more likely to be attributable to a source partner with a CD4 count <200 cells per μL at ART initiation than acquisitions in women. In Zimbabwe, a lower percentage of transmissions were attributable to a source partner who had received ART for fewer than 6 months since first initiation (range 11·0–39·4% in 2023), and similar patterns were seen with CD4 cell counts at re-initiation of ART after interruption.

EMOD (with outputs for Malawi) and HIV Synthesis (with outputs for Malawi, Zimbabwe, and South Africa) were able to define treatment interruptions among source partners as either first interruptions or subsequent interruptions (appendix p 13). According to EMOD projections, in 2023, among all transmissions arising from individuals who had interrupted treatment, 55·9% of transmissions in 2023 among individuals aged 15–24 years were attributable to a source partner who had interrupted treatment for the first time. This percentage decreased with age; 51·5% of transmissions among people aged 25–34 years and 35·7% of transmissions among people older than 35 years were attributable to a source partner who had interrupted treatment for the first time. In 2040, the proportion of people transmitting HIV after a subsequent interruption in their treatment increased compared with 2023. HIV Synthesis found no difference by age or calendar year in any setting—transmissions attributable to people who had interrupted treatment were most likely due to first interruptions in treatment.

## Discussion

With six well established, independently calibrated HIV transmission dynamics models, we aimed to identify at which stages in the HIV treatment cascade most transmissions occur. Modelled results differed across countries, and estimates for some stages of the HIV treatment cascade varied widely among the models. Although some models suggested that most transmissions were attributable to people living with HIV who were undiagnosed (particularly in Malawi and Zimbabwe), new transmissions were not primarily attributed to any single treatment state of source partners in other models but were instead attributable across a range of states. Trends in the distribution of HIV transmissions were generally predicted to be stable from 2023 to 2040, again spread across source partners who were undiagnosed, partners receiving ART with detectable viral loads, and (particularly in South Africa) partners who had interrupted ART. Across the three settings, most models found only very small proportions of transmissions attributable to people who had been diagnosed but had not yet started ART. One model, Optima-HIV, was able to estimate the percentage of transmissions attributable to individuals with low CD4 cell counts at the time of transmission. Fewer than half of individuals transmitting HIV had CD4 counts <200 cells per μL across the three settings in 2023, although this did vary by setting, with Malawi having the highest percentage of people with low CD4 cell counts at time of transmission (approximately 50%). This finding reflects the very high rates of diagnosis and treatment coverage in the model calibration, with a larger proportion of the small number of remaining HIV transmissions being attributed to individuals who had either remained undiagnosed or had experienced treatment failure and advanced HIV.

A recent study by Edun and colleagues,^7^ which used PHIA data across 14 countries in the ECSA region to identify sources of transmissions, showed that undiagnosed individuals contributed most to HIV transmission, although this proportion did vary across settings (proportions of transmissions from this group ranged from 52% in Eswatini in 2016-17 to 92% in Ethiopia in 2017-18).^7^ Another modelling study published in 2024 also showed that undiagnosed people living with HIV are likely to continue to contribute considerably to new HIV infections over the short term in Côte d’Ivoire, Mali, and Senegal (with a transmission population-attributable fraction for all people living with HIV with undiagnosed infection >60%).^18^ In our study, in Malawi and Zimbabwe in particular (but also in South Africa according to HIV Synthesis), some models also found a substantial proportion of transmissions attributable to undiagnosed individuals. However, the magnitude of the proportion of transmissions attributed to undiagnosed people was generally lower in our modelled results than in Edun and colleagues’ PHIA study, perhaps partly due to individuals’ undiagnosed statuses being established through self-reported knowledge of HIV status in the PHIA analyses.

Our individual model outputs have not been presented with uncertainty ranges due to the already extensive volume of model output data analysed; adding such ranges would make readability difficult. However, structural uncertainty has been considered implicitly through the inclusion of multiple models in this analysis. Our modelled results were heavily dependent on model assumptions and structure, and assumptions around transmission were inherent characteristics of the models themselves. We did not attempt to align models on either their structures or their underlying assumptions as we wanted the outputs to reflect the range of modelling approaches used in other contexts. By doing so, we were able to present the degree of structural uncertainty reflected by the six different model structures. Our results therefore reflect different potential scenarios within each of the three settings, which might explain their variability. For example, in Zimbabwe, the number of new infections was considerably lower according to the Optima HIV model than the HIV Synthesis model. Optima HIV also assumed higher testing rates than HIV Synthesis, such that the proportion of people living with HIV who were projected to know their status was close to 100%. That the proportion of new transmissions attributed to a source partner with undiagnosed HIV was considerably lower in Optima HIV results than the HIV Synthesis results was therefore unsurprising (as were the inverse results for transmissions from a partner receiving ART).

Transmissions among older individuals, in all countries modelled, were generally more likely to come from individuals who had interrupted ART. If interruption rates are assumed to be constant with respect to age (as they were with most of the models) and we can loosely assume that source partners are also likely to be older as a result of model age and sex mixing patterns, this finding can probably be explained by proportions of undiagnosed and ART-naive individuals declining with age, resulting in older people living with HIV having more time to be diagnosed and treated—and by implication, more time to have interrupted treatment. However, there is evidence to suggest that interruptions are less frequent at older ages,^19,20^ and this factor is being considered in future iterations of some of the models involved in these analyses (eg, Thembisa and Optima HIV). Some models did find male-to-female transmissions were more likely to be attributable to undiagnosed individuals than female-to-male transmissions. This is well evidenced; UNAIDS estimates suggest that a lower proportion of men are aware of their status compared with women.^21^

Albeit nuanced by age and sex differences across settings, the treatment status of source partners in South Africa was notably different to those seen in Malawi and Zimbabwe. In South Africa, models were mostly in agreement that a large proportion of transmissions (>50% according to Optima HIV and Thembisa) were attributable to a source partner who had interrupted treatment. This finding is in line with the lower proportion of people diagnosed with HIV receiving ART observed in South Africa (81%) than in Malawi (96%) and Zimbabwe (99%) and our modelled results (figure 1). Interruption rates in South Africa are high, the reasons for which are complex (including mobility issues, side-effects of treatment, little time to attend appointments, and stigma).^22^ In models, these rates are conveyed by assumptions regarding the number and duration of interruptions. However, rates of ART interruption and resumption are difficult to estimate reliably. In the absence of unique patient identifiers and electronic medical record systems that can be used to link patients across facilities, establishing how many individuals who are lost to follow-up resume ART at other facilities or what proportion of so-called gaps in care represent true ART interruptions as opposed to poor ART adherence or temporary receipt of ART from other facilities is almost impossible.

Interventions are needed across the HIV treatment cascade to reduce the risk of HIV transmission. At least 20% of transmissions across the three settings (and up to 70% in Zimbabwe) are attributable to source partners being undiagnosed. By increasing diagnostic testing, interventions have the potential to considerably affect the number of new transmissions. We have shown that a fair proportion of transmissions are attributable to source partners receiving ART, which might be a consequence of model assumptions around low numbers of new infections or high diagnosis rates resulting in scenarios in which nearly all people living with HIV receive ART. However, other explanations should also be considered. For example, transmissions attributable to source partners receiving ART are more common among older people and might suggest suboptimal adherence to treatment. The different models differ in their structure in relation to age and viral suppression. In HIV Synthesis, older people are assumed to have better adherence profiles, resulting in higher probabilities of viral suppression. In most other models, rates of viral suppression are not assumed to differ by age, although older people are more likely to be diagnosed and receive treatment for longer, resulting in higher viral suppression rates.

As countries are moving towards achieving the 95-95-95 HIV testing, treatment, and viral suppression targets, the proportion of transmissions attributable to individuals receiving ART will probably also increase. There is a need for increased viral load monitoring to ensure treatment success is measured at the individual level and that national HIV treatment goals can also be achieved.^23^ Point-of-care viral load assays have the potential to increase viral load coverage and can be used to identify people living with HIV who might benefit from adherence counselling, which has been shown to be an effective intervention in achieving an undetectable viral load.^24^

Transmissions attributable to individuals who had interrupted ART are also considerable, particularly in South Africa. Although interventions focused on educating people about the consequences of treatment interruption are likely to have an effect on transmissions, a deeper understanding is needed as to why interruptions happen. The reasons behind treatment interruptions are probably multifold and require wider interventions. These could include, for example, extending clinic hours, differentiated service delivery models, and campaigns to reduce stigma.^25,26^ There could also be substantial benefits from interventions aimed at re-engaging people with interrupted treatment, such as those that trace people lost to follow-up.^27^

There are limitations to our analyses. Modelling studies are reliant on empirical data, and in this case, the absolute number of new infections across settings will affect the model results presented. Our analysis was useful in highlighting that the different model estimates of the number of new HIV infections did not align well. This difference might have been due to several factors—eg, empirical incidence estimates typically have wide 95% CIs, hence model calibration might not have been closely aligned. Although models would have incorporated uncertainty around parameter estimates (different parameter values for each model are provided in the appendix [p 17]), differences between models could also be due to model assumptions (ie, which data sources to include and prioritise in the calibration process). All models had different underlying structures and could have been prone to model mis-specification bias.

This study has advanced the HIV modelling field. We found quite large differences in findings between the different models used, particularly in earlier iterations of outputs (not presented here). These initial differences led to our modelling teams reflecting on the assumptions that could explain these differences and modifying the models where possible. Moving forward, modelling teams will continue to collaborate and reassess assumptions allowing continuous model development, highlighting the need for modelling consortia. Without such consortium funding, multimodelling projects would be difficult.

In summary, across six independent and well established HIV epidemiological models set in the ECSA region, sources of transmission were found to span the HIV treatment cascade, with variation across settings partly explained by initial assumptions about the number of new infections and testing rates. Local data on these key outputs are needed to maximise the potential of modelling analyses, and customised analysis of HIV treatment gaps is needed to maximise HIV treatment as prevention.

## Supplementary Material

Suppl Materials

## Figures and Tables

**Table:** Key characteristics of the six contributing models

	Goals	Optima HIV	HIV Synthesis	EMOD	Thembisa	PopART-IBM
Modelled countries	Malawi, Zimbabwe, and South Africa	Malawi, Zimbabwe, and South Africa	Malawi, Zimbabwe, and South Africa	Malawi	South Africa	Zimbabwe
Structure	Compartmental model with disaggregation by sex and risk group; 1-year time step	Compartmental model with population disaggregation depending on setting (including sex, age, and risk group); 2·4-month time step	Individual-based stochastic model; 3-month time step	Network individual-based stochastic model; daily time step	Compartmental model with disaggregation by sex, age, risk group, and marital status; 1-month time step	Individual-based stochastic model with dynamic sexual network; timestep of 1/48 of a year
Approach to data calibration and estimates for specific settings	Epidemiological parameters (ie, probability of transmission per act and variation by stage of infection, presence of other STIs, and effectiveness of condoms and ART) are varied to fit the model to prevalence estimates from surveillance and surveys	Epidemiological parameters (ie, probability of transmission per act and variation by stage of infection [informed by CD4 cell counts and viral load monitoring], HIV testing rate, mortality rate, presence of other ulcerative STIs or tuberculosis, and effectiveness of condoms, circumcision, and unsuppressive ART) are varied to fit the model to prevalence estimates from surveillance and surveys	Parameters relating to population characteristics, sexual behaviour (eg, condomless sex) and age–gender mixing, HIV acquisition, HIV testing, natural history (ie, CD4 cell count and viral load), ART, risk of advanced HIV, and death are varied within plausible bounds to create a range of setting scenarios from which ones with outputs corresponding to observed data are selected	The model is parameterised with demographic data (including population size, fertility, and mortality) and age-specific and sex-specific epidemiological data (including prevalence, incidence, ART coverage, and voluntary male circumcision coverage); calibration was performed with a parallel simultaneous perturbation optimisation algorithm; roulette resampling in proportion to the likelihood of each simulation was used to select 250 model parameter sets	HIV transmission rates per sex act, HIV survival parameters, sexual behaviour parameters, HIV testing rates, and ART initiation rates are varied in calibrating the model to South African HIV prevalence data (from antenatal, household, and key population surveys), mortality data, HIV testing history data, ART programme data, and paediatric HIV data following a Bayesian approach	The model is parameterised with age-specific and sex-specific data on sexual partnerships and HIV prevention, testing, and treatment uptake from a population cohort in Manicaland, Zimbabwe; demographic data (including population size, fertility, and mortality) come from national estimates; the model was calibrated as follows: first, Latin hypercube sampling was used to generate N=100 000 parameter sets; the resulting runs were then target-fitted to national data on HIV prevalence for Zimbabwe at four timepoints stratified by sex, producing 10 model parameter sets consistent with all data
Sexual behaviour	Behaviours (eg, number of partners, sexual acts per partner, condom use, and needle sharing) differ by risk group (ie, female sex workers, male clients of sex workers, men and women with non-regular sex partners, faithful couples, men who have sex with men, and people who inject drugs)	Behaviours (eg, type of sexual partner [regular, casual, or commercial] and injecting), acts per partner, condom use, and needle sharing differ by age and risk group (ie, female sex workers, clients of sex workers, men who have sex with men, and people who inject drugs)	Two types of condomless sexual partnership are differentiated (long-term and short-term); long-term partnerships are remembered over time; rates of condomless sexual contact depend on age, type of partnership, and gender; men who have sex with men and people who inject drugs are not explicitly modelled	Four types of sexual partnership (ie, marital, informal, transitory, and commercial) are formed according to specifiable partner age patterns and are tracked over time	Three types of sexual partnership (ie, marital or cohabiting, short-term, and commercial) are differentiated, with different sexual frequencies and condom use for each; rates of short-term and commercial sex contact depend on risk group (high or low), age, marital status, and sex; there are separate assumptions for men who have sex with men	Two types of sexual partnership (casual and long-term) are differentiated, each with its own specific probability of condom use; the population is subdivided into three risk groups, with higher-risk individuals having higher rates of partner acquisition; partnerships are formed on the basis of risk-specific and age-specific partnership formation rates and an age-mixing matrix (by sex) derived from a Manicaland general population survey
Changes to sexual behaviour after diagnosis or starting ART	No changes to sexual behaviour are assumed as a result of diagnosis or starting ART	No changes to sexual behaviour are assumed as a result of diagnosis or starting ART	A reduction in condomless sex is assumed after diagnosis; no change in sexual behaviour is assumed from ART initiation to viral suppression	No changes to sexual behaviour are assumed as a result of diagnosis or starting ART	An increased frequency of sexual activity (due to increased CD4 cell counts) and higher levels of condom usage are assumed after diagnosis or starting ART	No changes to sexual behaviour are assumed as a result of diagnosis or starting ART
HIV acquisition determinants (including prevention interventions)	Acquisition risk depends on the characteristics of the individual (ie, number of partners, circumcision status [for male individuals], and PrEP use), the partner population (ie, HIV prevalence, ART use, and stage of infection), and the partnerships (ie, sexual acts per partner, prevalence of other STIs, type of sexual acts, and condom use)	Acquisition risk depends on the characteristics of the individual (ie, number of partners and number of injections), and the partnerships (ie, type of partner, sexual acts per partner, condom use, circumcision status [for male individuals], vertical transmission status, PrEP and PEP use, opiate substitution therapy use, and receptive needle sharing), and HIV status by population (ie, HIV testing, HIV diagnosis, HIV prevalence, unsuppressive ART use, and stage of infection)	Acquisition risk depends on the characteristics of the individual (ie, age, sex, number of condomless sexual partners, circumcision status [for male individuals], and PrEP use), the partner population (ie, HIV prevalence and viral load), and the partnership (ie, type of condomless sex partnership); transmission of HIV with specific drug resistance mutations is also modelled	Acquisition risk depends on the characteristics of the individual (ie, number of sexual partners, circumcision status [for male individuals], condom use, other STIs, and sexual acts per partner) and the partner (ie, HIV prevalence, ART use, and stage of infection)	Acquisition risk per sexual act depends on the characteristics of the individual (ie, age, sex, risk group, type of relationship, circumcision status [for male individuals], and PrEP use) and the characteristics of partners with HIV (ie, condom use, acute infection, CD4 cell count, ART status, and viral suppression)	Acquisition occurs only in serodiscordant partnerships; HIV acquisition can occur at each timestep in a serodiscordant partnership (ie, per 1/48 of a year) and depends on whether condoms are used in the partnership, whether the HIV-negative individual is taking PrEP (and circumcision if male), whether the HIV-positive partner is receiving ART, the stage of infection (ie, acute or CD4 stage if chronic), and the set-point viral load of the HIV-positive partner
HIV natural history	Rate of decline of CD4 cell count when not receiving ART depends on current CD4 cell count and age; mortality when not receiving ART depends on sex, age, and CD4 cell count	Rate of decline of CD4 cell count and viral load when not receiving ART depends on current CD4 cell count and viral load; mortality when not receiving ART depends on current CD4 cell count	Rate of decline of CD4 cell count is dependent on current viral load; viral load increases over time; risk of advanced HIV and death is dependent on current CD4 cell count, current viral load, age, and current use of co-trimoxazole	HIV prognosis is calculated with a Weibull distribution; the parameters of the distribution are derived from CD4 cell count and age at the time of infection; CD4 cell count decreases from the time of infection when not receiving ART	After the acute infection phase, individuals progress through four CD4 stages in the absence of ART, with rates of CD4 progression depending on age and sex; untreated mortality depends on age, sex, and CD4 cell count	After the acute infection phase, individuals not receiving ART progress through four CD4 stages, with rates of CD4 progression depending on the set-point viral load of the individual; untreated advanced HIV-related mortality occurs when the CD4 count is <200 cells per μL
HIV testing or diagnosis	Testing is by modality and population group and establishes knowledge of HIV status, but this is not linked to transmission since ART coverage is the direct input	Testing is modelled by modality, population group, and year, which establishes knowledge of HIV status and allows linkage to care and initiation of ART on the basis of treatment coverage level	Whether a person is tested or not is defined in each period; testing is indicated in antenatal clinics (for potential symptoms of HIV and potentially every 6 months for female sex workers); general testing with various degrees of targeting of people with higher probabilities of infection (dependent on testing history)	HIV testing or diagnosis occurs voluntarily, at antenatal visits, or once individuals are symptomatic	Includes five types of testing in adults: antenatal testing, testing in patients with opportunistic infections, general testing, self-testing, and index testing (ie, following referral by an HIV-positive partner); general testing rates vary by age, sex, and testing history	Testing is either general testing or symptomatic testing; general testing rates vary by sex and age and over time, and are capped to not exceed 3 million tests per year from 2021 onwards
ART	Risk of mortality while receiving ART is determined by age, sex, CD4 cell count at treatment initiation, and duration on treatment	Mortality while receiving ART depends on current CD4 cell count and ART status (ie, suppressive or unsuppressive)	Specific drugs, their current level of activity given drug resistance, and current ART adherence determine viral load, CD4 cell count change, and risk of further resistance; currently receiving ART has an independent effect on risk of advanced HIV and death over and above these factors	Risk of mortality while receiving ART is determined by age, sex, CD4 cell count at treatment initiation, and duration on treatment	Risk of mortality while receiving ART depends on baseline CD4 cell count, ART duration, age, and sex	Individuals receiving ART and who are virally suppressed do not experience further disease progression or advanced HIV-related mortality; those receiving ART but who are unsuppressed progress at a reduced rate 0·5–1 times that of unsuppressed individuals; risk of advanced HIV-related mortality while receiving ART is zero if virally suppressed and only occurs for individuals who are not receiving ART or who are receiving ART but are not virally suppressed when CD4 count <200 cells per µL, for which it is dependent on set-point viral load and sex
ART interruption	Individuals return to a CD4 cell count one category higher than at time of treatment initiation; survival progression is identical to individuals who are naive to treatment	CD4 cell count decreases following dropout from ART; prognosis is recalculated on the basis of age and CD4 cell count at the time of dropout; computation of ART prognosis after re-enrolment on treatment is the same as the initial enrolment with prognosis parameters corresponding to age and CD4 cell count at re-enrolment	Immediate (within 3 months) viral load return to pre-ART level, substantial initial decline in CD4 cell count towards pre-ART nadir	CD4 cell count decreases following dropout from ART; prognosis is recalculated on the basis of age and CD4 cell count at the time of dropout; computation of ART prognosis after re-enrolment is identical to the initial enrolment with prognosis parameters corresponding to age and CD4 cell count at re-enrolment	Immediate return of CD4 cell count and viral load to levels at the time of ART initiation after an ART interruption; however, no effect of ART interruption on mortality is modelled	HIV progression restarts at the CD4 cell count at the time of treatment initiation and with disease progression and advanced HIV-related mortality identical to individuals who are treatment naive; individuals can restart ART in future

ART=antiretroviral therapy. PEP=post-exposure prophylaxis. PrEP=pre-exposure prophylaxis. STI=sexually transmitted infection.

## Data Availability

No new primary data were collected for this modelling study. Source code for the models can be found on the individual model websites.

## Research in context

### Evidence before this study

In 2022, UNAIDS estimated 450 000 new HIV infections in east and southern Africa and 190 000 new HIV infections in west and central Africa. Identifying where new HIV transmissions are occurring in the HIV treatment cascade is key to reaching individuals with interventions to reduce the likelihood of onward HIV transmission. On June 20, 2024, we searched Web of Science with the search terms (“HIV”) AND (list of all countries in sub-Saharan Africa) AND ((“transmission”) AND (“acquisition”)) OR (“new infections”) to identify all articles investigating the proportion of HIV infections transmitted by individuals in different stages of the HIV treatment cascade. Two articles from 2024 investigating a similar question were identified. One of these studies, which used Population-based HIV Impact Assessment (PHIA) self-reported data, reported that most HIV transmissions were attributable to an undiagnosed source partner. However, PHIA data are largely reliant on self-reported HIV diagnosis and are cross-sectional so trends over time cannot be ascertained. The second study, a modelling study focusing on Côte d’Ivoire, Mali, and Senegal, also showed that a large proportion of new HIV infections could be due to undiagnosed people living with HIV.

### Added value of this study

This study was a joint modelling analysis conducted with six independent, calibrated, and well established models comparing trends over time in sources of HIV transmission, with a focus on Malawi, Zimbabwe, and South Africa. The results of this study show that interventions to reduce HIV transmission need to span across the HIV treatment cascade, focusing on undiagnosed individuals and individuals who had interrupted treatment.

### Implications of all the available evidence

There is a continued need for interventions focusing on HIV testing and treatment retention for maximum impact in reducing the number of new HIV transmissions. For individuals receiving treatment, these interventions should include increased viral load monitoring to identify people living with HIV who might benefit from adherence counselling and increased understanding of the reasons behind treatment interruptions.
